# Extended Hematological Parameters and Short-Term Mortality in Sepsis Patients: A Retrospective Study

**DOI:** 10.3390/jcm14093243

**Published:** 2025-05-07

**Authors:** Piotr F. Czempik, Agnieszka Wiórek

**Affiliations:** 1 Department of Anesthesiology and Intensive Care, Faculty of Medical Sciences in Katowice, Medical University of Silesia, 40-752 Katowice, Poland; 2 Transfusion Committee, University Clinical Center of Medical University of Silesia in Katowice, 40-752 Katowice, Poland

**Keywords:** complete blood count, intensive care unit, mortality, nucleated red blood cell, prognosis, sepsis

## Abstract

**Background/Objectives**: Sepsis has a high mortality rate, with septic shock exceeding 50%. The most common score for diagnosis and prognosis in sepsis is the Sepsis-related Organ Failure Assessment (SOFA). This study aimed to identify hematological parameters for the prediction of intensive care unit (ICU) mortality. **Methods**: This study collected demographic and clinical data from sepsis patients, including age, sex, infection site, length of stay, the SOFA, and ICU mortality. The standard laboratory panel included, among others, complete blood count with differential and reticulocyte panel. Intergroup differences were analyzed using Student’s *t*-test, Mann–Whitney U test, Pearson’s χ^2^, and Fisher’s test where applicable. The AUROC was used for evaluating the predictive value of hematological parameters. Multiple logistic regression was performed to exclude the impact of potential confounding factors. **Results**: This study analyzed data from 190 sepsis patients hospitalized in the ICU, excluding patients with other conditions significantly affecting mortality. The median age was 65.0 (IQR 51.0–71.0) years. The sexes were almost perfectly balanced. The sites of infection were mostly the abdominal cavity, lungs, and urinary tract. The short-term mortality rate was 30%, with higher SOFA scores and significant differences in leukocyte, platelet, and erythrocyte parameters between survivors and non-survivors. The percentage of nucleated red blood cells (NRBCs) showed the highest AUROC. **Conclusions**: The only reliable hematological parameter for predicting ICU mortality in sepsis patients may be the percentage of NRBCs. The presence of NRBCs in a blood smear is linked to a worse prognosis.

## 1. Introduction

Despite tremendous progress being made in its management, sepsis constitutes a significant healthcare problem. In 2017, there were approximately 48 million cases of sepsis and 11 million sepsis-related deaths globally [1]. According to a wide consensus, sepsis has been defined as “life-threatening organ dysfunction caused by dysregulated host response to infection” [2]. Sepsis is considered a medical emergency because its prognosis very much depends on the speed of diagnosis and adequate early management [3]. Despite enormous progress being made in the management of sepsis, it is associated with high mortality, with the mortality rate in a septic shock reaching more than 50%, especially in elderly patients [4,5]. By comparison, the mortality rate in myocardial infarction with ST-segment elevation is currently at about 8%, ranging from 4% to 15% in recent years [6,7,8]. Predictive scores have been developed for both the diagnosis and prediction of sepsis. Numerous scoring systems for the severity of illness include abnormalities in the complete blood count, although white blood cell and erythrocyte parameters are mostly under-represented. The most frequently used score for both the diagnosis and prediction of sepsis is the Sepsis-related Organ Failure Assessment (SOFA) [9]. Its abbreviated form is called “quick SOFA score” (qSOFA) and is used to improve the diagnosis of sepsis in hospital departments outside intensive care units (ICUs) [10]. The quick SOFA score is also used to identify patients at increased mortality risk and requiring ICU hospitalization for more than 72 h. The predictive value of an original SOFA score and its abbreviated form for diagnosing sepsis is not excellent. It has been shown that the area under the receiver operating characteristic (ROC) curve (AUROC) for the SOFA in ICUs and for qSOFA outside ICUs for the prediction of sepsis is approximately 0.80 [11].

Complete blood count with differential (CBC w/diff) is a standard, inexpensive laboratory test that aids in determining a patient’s immune competency and response to infection [12]. Nucleated red blood cells (NRBCs) are precursors of erythrocytes. They rarely appear in healthy adults’ blood smears but, if present, are considered a credible extended inflammatory parameter for outcome prediction [13].

This study aimed to search for hematological parameters that could predict short-term sepsis-related mortality.

## 2. Materials and Methods

This retrospective cohort study was carried out in a mixed medical–surgical ICU in a large academic hospital with 663 hospital beds, and the analyzed period was from September 2021 to March 2025. All patients diagnosed with either sepsis or septic shock were analyzed. Sepsis and septic shock were diagnosed using the most recent international definitions [2]. The modern definitions of sepsis or septic shock do not involve laboratory determinations [14]. We analyzed patients with sepsis and septic shock as a single group, as the primary factor contributing to mortality in both conditions is acute organ dysfunction caused by the host’s response to infection. Patients with additional diagnoses that could have an impact on mortality were excluded: non-escalation of therapy due to futility, sudden cardiac arrest, brain death, acute liver failure, etc. Only sepsis-related short-term (ICU) mortality was of interest to us. Complete blood counts with differential and reticulocyte parameters were compared between survivors and non-survivors. All data regarding demographics, clinical features, and laboratory parameters were taken from electronic medical records (AMMS, Asseco Medical Management Solutions, Rzeszów, Poland).

Demographics and clinical features included sex, age, primary infection site, time interval between the moment of ICU admission and collection of blood for hematological parameters, length of stay (LOS) in the ICU, mortality in the ICU, and SOFA score.

In the local ICU, the standard laboratory panel for sepsis patients includes the following: complete blood count with differential (CBC w/diff), reticulocyte panel (reticulocyte populations, reticulocyte maturity index, reticulocyte hemoglobin equivalent RET-He), PCT, C-reactive protein (CRP), creatinine, blood urea nitrogen (BUN), estimated glomerular filtration rate (eGFR), and total bilirubin. The hematological parameters were determined using a hematology analyzer (XN-1000, Sysmex, Kobe, Japan). A complete diagnostic panel in patients with sepsis requires the collection of blood for the following test tubes: an ethylenediaminetetraacetic acid (EDTA) tube, sodium citrate tube, and clot activator tube. Blood was collected from an artery through a cannula (BD Arterial Cannula, Becton, Dickinson and Company, Franklin Lakes, NJ, USA) using a vacuum system (BD Vacutainer^®^, Franklin Lakes, NJ, USA). Care was taken to not pollute blood samples with unfractionated heparin (UFH) from the flashing solution of the arterial line system. Blood samples were collected directly following admission to the ICU.

Statistical analyses were carried out with the use of licensed statistical software (18.0 Basic Edition, Stata, StataCorp LLC, College Station, TX, USA). Continuous variables were expressed as medians and interquartile ranges (IQRs, i.e., 25pc–75pc). Categorical variables were expressed as frequencies and percentages. The Shapiro–Wilk test and histograms were used to verify the type of distribution of continuous variables. Differences for continuous variables were assessed by independent samples Student’s *t*-test or the Mann–Whitney U test, depending on the type of variable distribution. Pearson’s χ^2^ test was utilized for categorical variables, with Fisher’s exact test used when it was necessary. The ROC curve was drawn for hematological parameters, and the AUROC was calculated to determine their value to predict short-term mortality. Multiple logistic regression was performed to exclude the impact of confounders (SOFA, age, sex). An ROC analysis was also carried out to assess the best cut-off values using the Liu method. The statistical tests were two-sided. Statistical significance was considered at *p* < 0.05.

The local bioethics committee approved this study. Because this study was retrospective in nature, the need for informed consent was waived. This study was performed according to the regulations of the Declaration of Helsinki. The results of this study were reported according to research reporting guidelines for observational studies (STROBE).

## 3. Results

**Figure 1:**
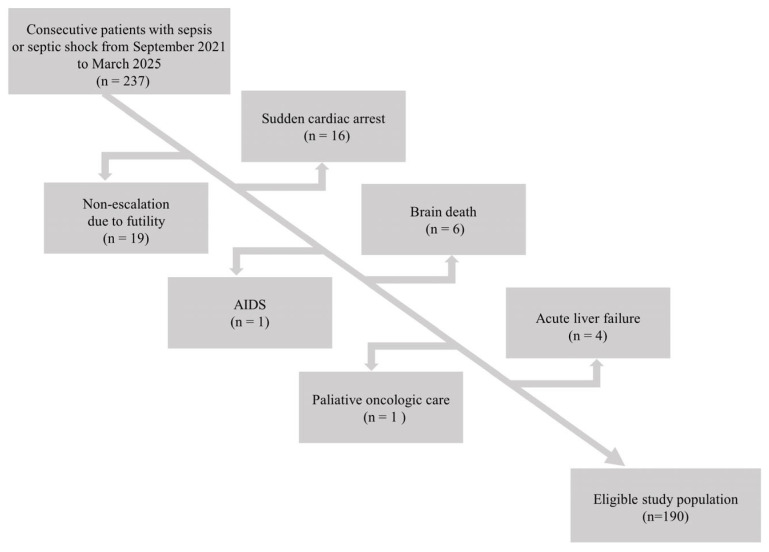
Study flow chart.

Data for 237 patients with sepsis admitted to the local ICU were taken from electronic health records. Patients with diagnoses other than sepsis that could have an impact on mortality were excluded. The flow chart for this study is presented in Figure 1.

Following exclusions, 190 patients were finally included in this study. The median age in the study group was 65.0 (IQR 51.0–71.0) years. There were 94 (49.5%) women and 96 (50.5%) men.

The majority of patients’ primary anatomical site of infection was intra-abdominal infection (*n* = 58, 30.8%), pneumonia (*n* = 57, 30.3%), or urinary tract infection (*n* = 43, 22.9%). The median values for biochemical parameters were as follows: PCT 3.68 (IQR 1.21–18.60) ng mL^−1^, CRP 177.5 (IQR 108.0–265.0) mg L^−1^, creatinine 1.19 (IQR 0.71–1.95) mg dL^−1^, blood urea nitrogen 30.0 (IQR 20.7–45.9) mg dL^−1^, and bilirubin 0.58 (IQR 0.30–1.00) mg dL^−1^. The median SOFA score in the cohort was 8.0 (IQR 6.0–11.0) points.

There were 130 (70.0%) survivors and 57 (30.0%) non-survivors. There was no association between patients’ sex and mortality (24.5 vs. 35.4% in females and males, respectively, *p* = 0.1). Non-survivors were older compared to survivors (68.0 (IQR 59.0–73.0) vs. 64.0 (IQR 46.0–71.0) years, *p* = 0.02). There were differences in biochemical parameters in survivors and non-survivors (Table 1).

The median SOFA score among survivors and non-survivors was 7.0 (IQR 5.0–10.0) and 11.0 (9.0–14.0) points, respectively (*p* < 0.001). There were significant differences between survivors and non-survivors in leukocyte parameters (monocyte percentage, monocyte number), platelet parameters (platelet number, platelet distribution width (PDW), mean platelet volume (MPV), platelet larger cell ratio (P-LCR)), and erythrocyte parameters (red blood cell distribution width (RDW), RDW standard deviation (RDW-SD), percentage of nucleated red blood cells (NRBCs), number of NRBCs). All aforementioned parameters were higher in non-survivors compared to survivors except platelet number and monocyte percentage and number (Table 2 and Table 3).

**Figure 2:**
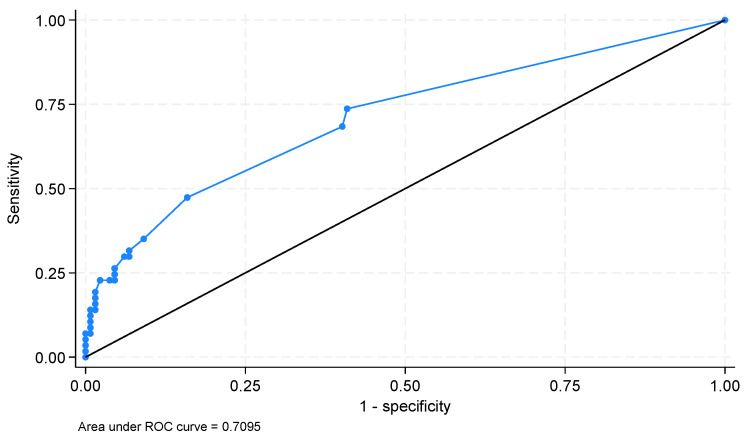
Area under receiver operating characteristic curve for percentage of nucleated red blood cells for prediction of intensive care unit mortality in sepsis patients.

The AUROCs for potential hematological parameters that could be used for ICU mortality prediction were as follows (only parameters with an AUROC > 0.500): PLT—0.607 (95%CI 0.516–0.697, *p* = 0.039), PDW—0.646 (95%CI 0.557–0.735; *p* = 0.002), RDW-SD—0.649 (95%CI 0.568–0.732; *p* = 0.002), MPV—0.656 (95%CI 0.568–0.743; *p* = 0.001), P-LCR—0.667 (95%CI 0.579–0.755; *p* < 0.001), number of NRBCs—0.708 (95%CI 0.625–0.791; *p* = 0.01), percentage of NRBCs—0.709 (95%CI 0.629–0.789; *p* = 0.006) (Figure 2).

The optimal cut-off for NRBCs was >0.02 × 10^3^ µL^−1^ (sensitivity 54.4%; specificity 84.1%); for the percentage of NRBCs, the cut-off was 0% (sensitivity 73.7%; specificity 59.1%).

We performed multiple logistic regression to exclude potential confounding factors that could impact the validity of NRBCs as a predictor of ICU mortality. NRBCs continued to be a statistically significant factor in the prediction of ICU mortality in the multiple logistic model. Other statistically significant variables were SOFA scoring and the age of the patients (Table 4).

## 4. Discussion

This study aimed to search for hematological parameters to predict sepsis-related ICU mortality. Relatively well-known and seemingly simple, routinely applied laboratory tests, such as blood count, have some untapped potential for patient diagnostics, with parameters that may be primarily overlooked in daily clinical practice. Complete blood count is the most commonly ordered hematological laboratory test. However, its value is limited, as it only gives information on the quantity of major essential blood components provided by automatic, algorithm-based analyzers. To better determine the types and subtypes of blood cells, their size and volume distribution, maturity, and cell type ratios, the analysis needs to be expanded by adding differential smears [15].

The WBC count with differential determines the total number of leukocytes, alongside the enumeration and percentage of each subtype. Leukocytes are primary components of systemic defensive reactions towards pathogens and injury, resulting in infectious, inflammatory, and immune responses [12]. The WBC count with differential traditionally should be examined in patients presenting signs, symptoms, or conditions associated with infections, inflammation, bone marrow alterations, and altered immune competency, particularly in an attempt to discern between infection with inflammation and inflammatory state without concomitant infection [12].

Routine hematological parameters have been analyzed to state their potential as extended inflammatory parameters to determine timely sepsis diagnosis and prognostication [16]. In a recent study by Herawati et al., sepsis patients presented with varied activation stages of neutrophils and lymphocytes, with higher total white blood cell count, neutrophil count, and percentage relative to total WBC, and lower monocyte percentage relative to total WBC, compared to non-sepsis and healthy controls, which is partially in line with our findings [17]. Through logistic regression model analysis, the authors concluded that standard hematological results in combination with inflammatory parameters could aid in timely clinical decision-making in suspected sepsis cases without imposing additional burdens on patients. In our study, we showed that confounding factors, like SOFA scoring, age, or sex, did not have impact on the conclusions of our study.

Amundsen et al. discussed the role of NRBCs in evaluating the prognosis of patients with suspected sepsis and their role as a marker that would potentially be useful in the emergency department (ED) setting [18]. There is a constant need for reliable, time-saving, broadly available parameters during initial diagnostics in the ED environment. Timely recognition of patients’ probable deterioration should lead to timely admission to the ICU or any other proper hospital ward for treatment. NRBCs can be measured accurately by the most accessible analyzers, even at deficient concentrations, which makes them possible prognostic markers [19]. The authors evaluated patients with suspected sepsis, noticing increased 30-day mortality in patients with elevated NRBCs, with a higher number of NRBC-positive patients in a subgroup of patients in whom sepsis was confirmed. The study concluded that NRBCs add prognostic value to clinical scoring systems and standard laboratory tests in the ED, with the prognostic value of NRBCs being more significant than some components of the SOFA score [18]. In our study, we showed that the odds ratio (OR) for NRBCs in the prediction of ICU mortality was higher than the OR for the SOFA.

Purtle et al. analyzed the occurrence of the circulating NRBCs in the peripheral blood as an indicator of an extreme increase in erythropoietic activity, detected in stress states like inflammation, hematological malignancy, pathological hematopoiesis, massive hemorrhage, or severe hypoxia [20]. The researchers emphasized the need to craft and test new models that could predict post-discharge outcomes in ICU patients and ICU survivors, aimed at tailoring interventions and improving outcomes [21]. They attempted to determine if critically ill patients who manifested NRBCs have higher 90-day mortality following hospital discharge. Sepsis patients were isolated as a study subgroup, and sepsis events were included in the adjustment for the accuracy of statistical models. Overall, the elevated NRBCs were connected to a raised risk of hospital readmission and delayed mortality. Additionally, RDW was correlated with NRBC count [21].

Studies indicate that hematological parameters, including RDW, PDW, MPV, and NRBC counts, serve as key indices for bloodstream infections and sepsis-related acute organ failure. These indices offer diagnostic performance that is on par with procalcitonin and surpass CRP, similar to laboratory markers of renal and hepatic function [21,22,23].

Our study is not devoid of limitations. The obvious limitation of this study is its retrospective mature, and previously recorded data may lack completeness or accuracy. However, we have taken every effort to make sure that the data were as complete and accurate as possible. Another limitation of a retrospective study is the lack of a control group Yet another limitation is the fact that this was a single-center study, which may limit the generalizability of our findings to other settings. However, to increase the reliability of our results, we performed additional a posteriori power calculations regarding our study’s sample size for the study group. We discovered that we would require a minimum of 168 participants to confirm a significant between-group difference with an alpha of <0.05 and a beta of 0.20. We also performed a sample size calculation to verify the significance of the ROC analysis for the ICU mortality prediction. We discovered that we would require at least 147 participants to establish the significance of an AUROC value of at least 0.7, with a ratio of sample sizes between negative and positive groups of 2 to 1, an alpha of <0.001, and a beta of 0.20. Therefore, while we acknowledge that our sample size is small, it is sufficient to draw conclusions, and our study was in no way underpowered. We included more than the minimal number of subjects calculated for both statistical purposes.

## 5. Conclusions

Among different hematological parameters in sepsis patients, the only acceptable parameter for predicting ICU mortality is the percentage of NRBCs. The presence of NRBCs in a blood smear is associated with a worse prognosis in patients with sepsis.

## Figures and Tables

**Table 1:** Biochemical parameters in survivors and non-survivors of sepsis.

Parameter	Survivors [Me ^1^ (IQR ^2^)]	Non-Survivors [Me (IQR)]	*p*-Value
Procalcitonin	3.20 (1.12–16.50)	6.81 (1.89–28.10)	0.058
C-reactive protein	169.0 (108.0–263.0)	191.0 (117.0–300.0)	0.317
**Creatinine**	**0.99 (0.67–1.72)**	**1.66 (1.11–2.62)**	**<0.001**
**Blood urea nitrogen**	**26.66 (16.96–41.50)**	**41.99 (29.76–58.41)**	**<0.001**
**Bilirubin (total)**	**0.47 (0.27–0.83)**	**0.77 (0.48–1.94)**	**<0.001**

^1^ Median value, ^2^ interquartile range. In bold: statistically significant differences.

**Table 2:** Leucocyte and platelet parameters in survivors and non-survivors of sepsis.

Parameter	Non-Survivors [Me ^1^ (IQR ^2^)]	Non-Survivors [Me (IQR)]	*p*-Value
White blood cells [×10^3^ µL^−1^]	12.35 (8.55–20.98)	14.05 (6.15–20.98)	0.696
Lymphocytes [%]	6.1 (4.3–10.9)	6.8 (3.6–11.2)	0.821
Lymphocytes [×10^3^ µL^−1^]	0.82 (0.62–1.23)	0.72 (0.39–1.16)	0.098
**Monocytes [%]**	**5.8 (3.9–7.8)**	**3.8 (2.8–5.9)**	**0.001**
**Monocytes [**×10^3^ µL^−1^]	**0.73 (0.42–1.21)**	**0.59 (0.27–0.99)**	**0.041**
Neutrophils [%]	84.9 (76.9–87.8)	85.1 (78.2–89.6)	0.338
Neutrophils [×10^3^ µL^−1^]	10.4 (7.1–18.0)	11.7 (5.5–17.8)	0.802
Eosinophils [%]	0 (0–0.5)	0 (0–0.2)	0.131
Eosinophils [×10^3^ µL^−1^]	0.01 (0.0–0.05)	0.0 (0.0–0.03)	0.052
Basophils [%]	0.2 (0.1–0.3)	0.2 (0.1–0.3)	0.902
Basophils [×10^3^ µL^−1^]	0.02 (0.01–0.04)	0.02 (0.01–0.04)	0.854
Immature granulocytes [%]	1.3 (0.5–2.8)	1.5 (0.8–3.2)	0.201
Immature granulocytes [×10^3^ µL^−1^]	0.16 (0.05–0.49)	0.15 (0.06–0.57)	0.982
**Platelets [×10^3^ µL^−1^]**	**215 (150–298)**	**174 (112–245)**	**0.020**
**Mean platelet volume [fL]**	**10.5 (9.9–11.2)**	**11.0 (10.2–12.0)**	**<0.001**
Plateletcrit [%]	0.23 (0.16–0.31)	0.19 (0.14–0.29)	0.115
**Platelet distribution width [%]**	**11.6 (10.3–12.8)**	**12.9 (11.2–15.5)**	**0.002**
**Platelet larger cell ratio [%]**	**28.8 (23.6–34.5)**	**32.8 (27.0–41.2)**	**<0.001**

^1^ Median value, ^2^ interquartile range. In bold: statistically significant differences.

**Table 3:** Erythrocyte parameters in non-survivors and survivors of sepsis.

Parameter	Survivors [Me ^1^ (IQR ^2^)]	Non-Survivors [Me (IQR)]	*p*-Value
Red blood cells [×10^3^ µL^−1^]	3.33 (2.77–3.81)	3.29 (3.00–3.72)	0.576
Hemoglobin [g dL^−1^]	9.6 (8.4–11.6)	10.4 (8.8–11.5)	0.291
Hematocrit [%]	30.4 (25.9–34.7)	31.7 (26.8–35.1)	0.293
Mean cell volume [fL]	91.3 (87.7–95.9)	94.0 (89.5–98.7)	0.055
MCHC ^3^ [g dL^−1^]	32.5 (31.7–33.7)	32.8 (31.8–33.7)	0.718
Reticulocytes [‰]	16.8 (11.6–22.8)	15.2 (12.3–22.4)	0.519
**RDW ^4^ [%]**	**14.8 (13.7–16.8)**	**15.5 (14.6–17.1)**	**0.033**
**RDW-SD ^5^ [fL]**	**49.3 (45.9–54.5)**	**52.7 (49.6–57.1)**	**0.001**
**NRBCs ^6^ [%]**	**0.0 (0.0–0.1)**	**0.1 (0.0–0.7)**	**<0.001**
**NRBCs [×10^3^ µL^−1^]**	**0.0 (0.0–0.0)**	**0.0 (0.0–0.1)**	**<0.001**
Reticulocytes [×10^9^ L^−1^]	54.1 (39.5–77.6)	48.6 (35.7–87.4)	0.464
Immature reticulocyte fraction [%]	15.1 (10.3–23.8)	15.8 (10.7–24.7)	0.822
Low fluorescence ratio [%]	84.9 (76.1–89.7)	84.2 (75.3–89.3)	0.838
Medium fluorescence ratio [%]	12.3 (8.7–15.8)	12.4 (8.7–14.5)	0.615
High fluorescence ratio [%]	2.9 (1.3–8.5)	3.5 (1.5–9.3)	0.704
RET-He **^7^** [pg]	30.0 (26.9–32.8)	30.4 (27.2–32.8)	0.899

^1^ Median value, ^2^ interquartile range, ^3^ mean cell hemoglobin concentration, **^4^** red blood cell distribution width, **^5^** standard deviation of red blood cell distribution width, **^6^** nucleated red blood cell, **^7^** reticulocyte hemoglobin equivalent. In bold: statistically significant differences.

**Table 4:** Multiple logistic regression analysis for prediction of intensive care unit mortality in sepsis patients.

Parameter	Odds Ratio (95%CI ^1^)	*p*-Value
**Nucleated red blood cells (percentage)**	**1.75 (1.07–2.86)**	**0.025**
**Sepsis-related Organ Failure Assessment**	**1.22 (1.10–1.35)**	**<0.001**
**Age**	**1.03 (1.00–1.06)**	**0.049**
Male sex	1.46 (0.69–3.12)	0.323

^1^ Confidence interval. In bold: statistically significant differences.

## Data Availability

The data presented in this study are only available on request from the corresponding author due to privacy restrictions.

## References

[1] Rudd K.E., Johnson S.C., Agesa K.M., Shackelford K.A., Tsoi D., Kievlan D.R., Colombara D.V., Ikuta K.S., Kissoon N., Finfer S. (2020). Global, regional, and national sepsis incidence and mortality, 1990–2017: Analysis for the Global Burden of Disease Study. Lancet.

[2] Singer M., Deutschman C.S., Seymour C.W., Shankar-Hari M., Annane D., Bauer M. (2016). The Third International Consensus Definitions for Sepsis and Septic Shock (Sepsis-3). JAMA.

[3] Gyawali B., Ramakrishna K., Dhamoon A.S. (2019). Sepsis: The evolution in definition, pathophysiology, and management. SAGE Open Med..

[4] Bauer M., Gerlach H., Vogelmann T., Preissing F., Stiefel J., Adam D. (2020). Mortality in sepsis and septic shock in Europe, North America and Australia between 2009 and 2019—Results from a systematic review and meta-analysis. Crit. Care.

[5] Nasa P., Juneja D., Singh O. (2012). Severe sepsis and septic shock in the elderly: An overview. World J. Crit. Care Med..

[6] Choudhury T., West N.E., El-Omar M. (2016). ST elevation myocardial infarction. Clin. Med..

[7] Pascual I., Hernandez-Vaquero D., Almendarez M., Lorca R., Escalera A., Díaz R., Alperi A., Carnero M., Silva J., Morís C. (2020). Observed and Expected Survival in Men and Women After Suffering a STEMI. J. Clin. Med..

[8] Oraii A., Shafeghat M., Ashraf H., Soleimani A., Kazemian S., Sadatnaseri A., Saadat N., Danandeh K., Akrami A., Balali P. (2024). Risk assessment for mortality in patients with ST-elevation myocardial infarction undergoing primary percutaneous coronary intervention: A retrospective cohort study. Health Sci. Rep..

[9] Vincent J.-L., Moreno R., Takala J., Willatts S., De Mendonça A., Bruining H., Reinhart C.K., Suter P.M., Thijs L.J. (1996). The SOFA (Sepsis-related Organ Failure Assessment) score to describe organ dysfunction/failure. On behalf of the Working Group on Sepsis-Related Problems of the European Society of Intensive Care Medicine. Intensive Care Med..

[10] Fernandes S., Wyawahare M. (2020). Utility of quick sepsis-related organ failure assessment (qSOFA) score to predict outcomes in out-of-ICU patients with suspected infections. J. Fam. Med. Prim. Care.

[11] Seymour C.W., Liu V.X., Iwashyna T.J., Brunkhorst F.M., Rea T.D., Scherag A., Rubenfeld G., Kahn J.M., Shankar-Hari M., Singer M. (2016). Assessment of clinical criteria for sepsis: For the third international consensus definition for sepsis and septic shock (Sepsis-3). JAMA.

[12] George-Gay B., Parker K. (2003). Understanding the complete blood count with differential. J. Perianesth. Nurs..

[13] Pikora K., Krętowska-Grunwald A., Krawczuk-Rybak M., Sawicka-Żukowska M. (2023). Diagnostic Value and Prognostic Significance of Nucleated Red Blood Cells (NRBCs) in Selected Medical Conditions. Cells.

[14] Evans L., Rhodes A., Alhazzani W., Antonelli M., Coopersmith C.M., French C., Machado F.R., Mcintyre L., Ostermann M., Prescott H.C. (2021). Surviving Sepsis Campaign: International Guidelines for Management of Sepsis and Septic Shock 2021. Crit. Care Med..

[15] Rahmnitarini A., Hernaningsih Y., Indrasari Y.N. (2019). The stability of sample storage for complete blood count (CBC) toward the blood cell morphology. Bali Med. J..

[16] Ekici-Günay N., Çakır I., Çelik T. (2017). Is there clinical value in counting nucleated red blood cells and platelet indices in primary immunodeficiency disease?. Turk. J. Pediatr..

[17] Herawati S., Somia I.K.A., Kosasih S., Wande I.N., Felim J., Payana I.M.D. (2024). Integrating Routine Hematological and Extended Inflammatory Parameters as a Novel Approach for Timely Diagnosis and Prognosis in Sepsis Management. Diagnostics.

[18] Amundsen E.K., Binde C., Christensen E.E., Klingenberg O., Kvale D., Holten A.R. (2021). Prognostic Value of Nucleated RBCs for Patients With Suspected Sepsis in the Emergency Department: A Single-Center Prospective Cohort Study. Crit. Care Explor..

[19] Da Rin G., Vidali M., Balboni F., Benegiamo A., Borin M., Ciardelli M.L., Dima F., Di Fabio A., Fanelli A., Fiorini F. (2017). Performance evaluation of the automated nucleated red blood cell count of five commercial hematological analyzers. Int. J. Lab. Hematol..

[20] Danise P., Maconi M., Barrella F., Di Palma A., Avino D., Rovetti A., Gioia M., Amendola G. (2011). Evaluation of nucleated red blood cells in the peripheral blood of hematological diseases. Clin. Chem. Lab. Med..

[21] Purtle S.W., Horkan C.M., Moromizato T., Gibbons F.K., Christopher K.B. (2017). Nucleated red blood cells, critical illness survivors and postdischarge outcomes: A cohort study. Crit. Care.

[22] Tang W., Zhang W., Li X., Cheng J., Liu Z., Zhou Q., Guan S. (2020). Hematological parameters in patients with bloodstream infection: A retrospective observational study. J. Infect. Dev. Ctries..

[23] Zhang H.B., Chen J., Lan Q.F., Ma X.J., Zhang S.Y. (2016). Diagnostic values of red cell distribution width, platelet distribution width and neutrophil-lymphocyte count ratio for sepsis. Exp. Ther. Med..

